# Draft Genome Sequence of the *Pandoraea apista* LMG 16407 Type Strain

**DOI:** 10.1128/genomeA.01300-15

**Published:** 2015-11-12

**Authors:** A. D. Millard, L. F. Westblade, J. J. LiPuma, K. Vavikolanu, T. D. Read, M. J. Pallen, E. M. Burd, C. I. Constantinidou

**Affiliations:** a Microbiology and Infection Unit, Warwick Medical School, University of Warwick, Coventry, United Kingdom; b Department of Pathology and Laboratory Medicine, Weill Cornell Medical College, New York, New York, USA; c Department of Pediatrics, University of Michigan, Ann Arbor, Michigan, USA; d Department of Medicine, Department of Infectious Diseases, Emory University School of Medicine, Atlanta, Georgia, USA; e Emory Antibiotic Resistance Center, Atlanta, Georgia, USA; f Department of Pathology and Laboratory Medicine, Emory University School of Medicine, Atlanta, Georgia, USA

## Abstract

*Pandoraea* species, in particular *Pandoraea apista*, are opportunistic, multidrug-resistant pathogens in persons with cystic fibrosis (CF). To aid in understanding the role of *P. apista* in CF lung disease, we used Illumina MiSeq and nanopore MinION technology to sequence the whole genome of the *P. apista* LMG 16407^T^.

## GENOME ANNOUNCEMENT

The genus *Pandoraea* is a group of multidrug-resistant, nonglucose fermenting, Gram-negative rods that have been recovered from the respiratory tract of cystic fibrosis (CF) patients (1–3). Members of the genus are closely related to other CF pathogens, most notably *Burkholderia* species (1, 2). Therefore, accurate genus- and species-level identification can be challenging. *Pandoraea apista* is one of the most frequently isolated *Pandoraea* species from CF patients (3) and has been associated with transmission between patients (4). To better understand its pathogenic traits and assist phylogenetic analysis of the genus, we sequenced the genome of the type strain, *P. apista* LMG 16407^T^, originally isolated from a CF patient (1).

Bacterial genomic DNA was extracted using the DNeasy blood and tissue kit (Qiagen, USA) and sequenced using MiSeq (Illumina, USA) and MinION (Oxford Nanopore Technologies, UK) instruments. A total of 1 ng of genomic DNA was prepared using the Nextera XT DNA sample preparation kit (Illumina) before sequencing on the MiSeq platform using the paired-end 2 × 251-bp (version 2) protocol. Prior to analysis on the MinION instrument, 1 µg of unsheared genomic DNA was treated with the Genomic DNA sequencing kit (SQK-MAP-002; Oxford Nanopore Technologies).

Using SPAdes (5), data derived from both sequencers were assembled to produce a draft genome, with the options of -careful and -pacbio for the MinION reads. Only contigs longer than 500 bp and with greater than 5× depth of coverage were included in the analysis. To correct any miscalled bases, the assembled genome was reviewed for errors by mapping reads against the individual contigs with BWA-MEM using the default settings (6). Contigs were sorted against the *P. apista* TF81F4 genome (CP010518) using the Mauve aligner (7). The draft genome included 45 contigs greater than 500 bp with a N_50_ of 223,394 bp; the longest contig was 716,258 bp. The estimated size of the whole genome is 5,537,404 bp (mean depth of coverage, 39×) with a G+C content of 63%.

Genome annotation with Prokka (8) identified 4,993 potential protein-coding sequences (CDS), 7 rRNAs, and 62 tRNAs. The CDS include predicated virulence factors also present in the related genus *Burkholderia* (http://www.mgc.ac.cn/cgi-bin/VFs/compvfs.cgi?Genus=Burkholderia) and encompass genes associated with flagella, adhesion, capsule, and type III and type IV secretion systems. Additionally, the *P. apista* LMG 16407^T^ strain harbors a putative 61,143-bp plasmid that encodes a complete type IV secretion system*.*

Phylogenetic analysis of single copy genes present in the 20 publically available *Pandoraea* species genomes was performed using RAxML version 7.2.8 (9). The resultant data revealed that *P. apista* LMG 16407^T^ is part of a clade composed of *P. apista* strains TF81F3, TF80G25, and AU2161. Average nucleotide identity (ANI) analysis demonstrated that the sequenced *P. apista* strains have an ANI greater than 99%, which exceeds the 95 to 96% threshold used to define a species (10), and are therefore considered members of the same species.

### Nucleotide sequence accession number.

This draft genome sequence of *P. apista* LMG 16047^T^ has been deposited in DDBJ/ENA/GenBank under the accession number CEWL00000000.
